# Transmission Mode Predicts Specificity and Interaction Patterns in Coral-*Symbiodinium* Networks

**DOI:** 10.1371/journal.pone.0044970

**Published:** 2012-09-18

**Authors:** Nicholas S. Fabina, Hollie M. Putnam, Erik C. Franklin, Michael Stat, Ruth D. Gates

**Affiliations:** 1 Department of Evolution and Ecology, University of California Davis, Davis, California, United States of America; 2 Hawaii Institute of Marine Biology, School of Ocean and Earth Science and Technology, University of Hawaii, Kaneohe, Hawaii, United States of America; 3 The UWA Oceans Institute and Centre for Microscopy, Characterisation and Analysis University of Western Australia, Perth, Western Australia, Australia; 4 Australian Institute of Marine Science, The University of Western Australia, Perth, Western Australia, Australia; Leibniz Center for Tropical Marine Ecology, Germany

## Abstract

Most reef-building corals in the order *Scleractinia* depend on endosymbiotic algae in the genus *Symbiodinium* for energy and survival. Significant levels of taxonomic diversity in both partners result in numerous possible combinations of coral-*Symbiodinium* associations with unique functional characteristics. We created and analyzed the first coral-*Symbiodinium* networks utilizing a global dataset of interaction records from coral reefs in the tropical Indo-Pacific and Atlantic Oceans for 1991 to 2010. Our meta-analysis reveals that the majority of coral species and *Symbiodinium* types are specialists, but failed to detect any one-to-one obligate relationships. Symbiont specificity is correlated with a host’s transmission mode, with horizontally transmitting corals being more likely to interact with generalist symbionts. Globally, *Symbiodinium* types tend to interact with only vertically or horizontally transmitting corals, and only a few generalist types are found with both. Our results demonstrate a strong correlation between symbiont specificity, symbiont transmission mode, and community partitioning. The structure and dynamics of these network interactions underlie the fundamental biological partnership that determines the condition and resilience of coral reef ecosystems.

## Introduction

Scleractinian corals, the foundation of coral reefs, are extremely sensitive to global changes [1], [2]. The condition and function of the coral holobiont is intimately tied to the identity of its symbiotic partners, which enhance or constrain coral responses to environmental stressors. Thus, there is an increasing need to study the symbiome – the collection of species with enduring associations within the physical limits of the coral colony [3]. Temperature anomalies are generally believed to present the largest threat to coral persistence [4], as they cause corals to disassociate from their symbiotic partners, particularly when coupled with UV stress [5]. This process leads to coral bleaching and, in extreme or persistent cases, colony mortality.

Most scleractinian corals depend on endosymbiotic dinoflagellates in the genus *Symbiodinium* for nutrition and survival [6]. The coral-*Symbiodinium* association is considered mutualistic, with corals receiving photosynthetic carbon and *Symbiodinium* receiving nitrogen, phosphorus, and carbon dioxide. Indeed, *Symbiodinium* commonly provide a coral with over 90% of its energetic requirements. That said, associations can also be commensalistic, or even antagonistic, depending on partner identity and environmental conditions [7], [8], [9]. Complicating matters is the diversity present in both guilds: there are hundreds of coral species and *Symbiodinium* types, with known diversity rapidly increasing with each new field study [10]. Currently, there are nine recognized evolutionary lineages of *Symbiodinium*, clades A through I [11].

*Symbiodinium* clades are thought to have general functional characteristics that affect coral responses to a range of environmental variables. Clade B and D *Symbiodinium* are tolerant of temperature extremes [12], while clade A symbionts produce amino acids that protect against UV radiation [9], [13]. Clade D is considered the most tolerant of disturbances and is thought to thrive in marginal environments because of a weedy or opportunistic life history [14]. Indeed, corals with clade D are much more common in thermally disturbed reefs or pools [15], [16]. However, there appears to be a tradeoff between short-term responses to temperature anomalies and long-term survival. While clade D continues photosynthesis during and after temperature stress [17], the presence of clade C is correlated with higher relative growth and lower relative mortality, both in juvenile and adult corals [18], [19]. Understanding the functional differences between *Symbiodinium* and the implications for coral survival is critical to forecasting coral responses to the increases in anomalous ocean temperatures predicted with climate change.

The potential for corals to respond to environmental changes is a function of partner identity, thus the ability of corals to interact with functionally diverse assemblages of symbionts could enhance their resilience. This line of thinking led to the Adaptive Bleaching Hypothesis [20], [21], which posits that under adverse environmental settings, the ability to interact with diverse partners allows corals to shift (acquire new symbiont partners from the environment) or shuffle (adjust the relative abundances of existing partners) their symbiont assemblages to enhance performance [13]. Indeed, a diverse *Symbiodinium* and microbial assemblage provides more consistent and diverse services in anemones and sponges [22], [23].

On the other hand, corals with obligatory symbiotic relationships that are functionally superior may be more environmentally resilient. Corals transmit *Symbiodinium* either vertically, from parent colony to egg or brooded larvae, or horizontally, where propagules or new recruits acquire symbionts from the environment [24]. Vertical transmission is expected to increase symbiotic fidelity due to the coupling of coral and *Symbiodinium* fitness [25]. Thus, corals that transmit symbionts vertically may have more specialized and mutualistic *Symbiodinium*, while horizontally transmitted symbionts may be more generalized and antagonistic [26]. Despite the performance implications of coral specificity and transmission mode, no studies to date have explored the existing coral-*Symbiodinium* association data at multiple spatial scales for specificity or transmission trends.

The fitness of a coral is a function of its potential symbiotic partners, which in turn is a function of community patterns of interaction. To better understand the coral response to stress, it is necessary to address key questions related to the symbiotic unions including: Are corals predominantly specialists interacting with select *Symbiodinium* types, or generalists interacting with diverse groups of symbionts? Likewise, do *Symbiodinium* specialize or generalize in their partnerships with corals? Does symbiont transmission mode affect coral-*Symbiodinium* interaction patterns? One approach to addressing these complex yet fundamental questions in coral biology is network theory. Network theory is a framework for quantifying mutualistic interaction patterns [27], [28] and their relationship to community stability [29], [30], [31]. In mutualistic interaction networks, vertices represent species and edges represent the interaction between those species. Species are then connected if an interaction occurs between any individuals in their populations. We use network theory and general results from existing mutualistic network studies as an approach to understand coral responses to global changes, which are determined by these symbiotic characteristics [9], [20].

In this study, we explore coral-*Symbiodinium* associations using a long-term, global database of observed *Symbiodinium* associations. First, we characterize coral and *Symbiodinium* specificity at reef, ocean basin, and global scales. Second, we test whether transmission mode correlates with specificity of either host or symbiont. We additionally show how interaction patterns in coral-*Symbiodinium* networks compare to those in other mutualistic communities, and discuss their connection to community resilience (definitions reviewed in [32]).

## Results

**Figure 1:**
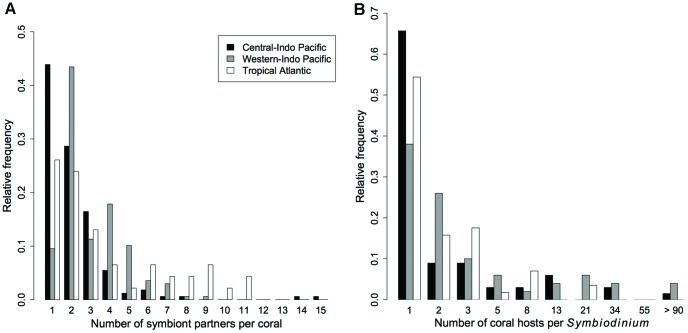
Degree (number of interactions) distributions for (a) coral and (b) *Symbiodinium* in the biogeographic realms of the central Indo-Pacific, western Indo-Pacific, and tropical Atlantic. Each group has many specialists and few generalists. *Symbiodinium* interaction numbers are logarithmically binned.

**Figure 2:**
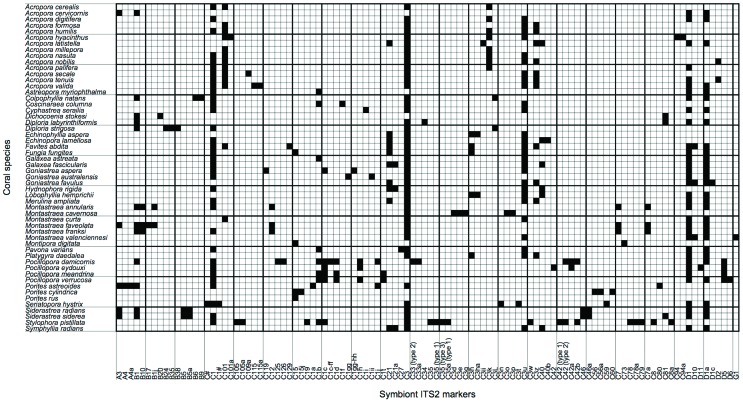
A matrix representation of the well-sampled global network of corals and *Symbiodinium*. Black or filled elements denote interactions that have been observed (e.g., between *Acropora cervicornis* and type A3), while white or empty elements denote interactions that have not been observed (e.g., between *Acropora cervicornis* and type A4).

**Figure 3:**
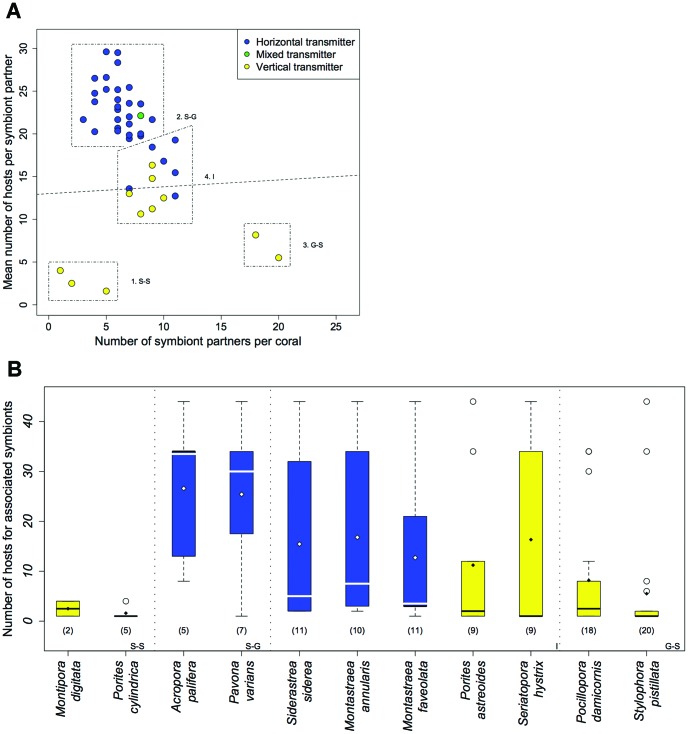
Relationships between symbiont transmission mode and specificity in the well-sampled global network of 54 coral species and 106 *Symbiodinium* types. (a) The number of interactions for coral species and the mean number of interactions for their *Symbiodinium* partners. The nearly horizontal, dashed line is the statistical partition between horizontally and vertically transmitting corals. K-means cluster numbering corresponds with text description. (b) Boxplot showing variance in symbiont specificity for well-sampled corals in each cluster. Each coral has at least 30 interaction records and colors are consistent with (a). The number of symbiont partners per coral are in parentheses.

Our analysis of coral-*Symbiodinium* interaction data revealed that there were many specialist coral species and *Symbiodinium* types and few generalists at reef, ocean basin, and global scales (Table 1, Fig. 1) (note that specialist and generalist are relative terms). We then restricted our analyses to the global network of 54 well-sampled corals and their 106 symbiont partners (Tables S1 and S2, Fig. 2; see methods). Grouping of corals into “specificity clusters” based on the number of interactions for a given coral and the mean number of interactions for its associated symbionts, resulted in four specificity clusters: (1) specialist corals with specialist *Symbiodinium*, (2) specialist corals with generalist *Symbiodinium*, (3) generalist corals with specialist *Symbiodinium*, and (4) intermediate corals with intermediate *Symbiodinium* (Fig. 3a). Corals in each cluster differed in the number of interactions for host and symbiont, as well as the variance in the number of interactions for associated symbionts (Fig. 3b). For example, corals in the specialist-generalist cluster associated with few *Symbiodinium* types, which interacted with many other hosts on average. Corals in the generalist-specialist cluster associated with many specialist symbionts, which had few alternative hosts. Corals in the former group interacted with both extreme specialists and extreme generalists, while corals in the latter group interacted only with specialists. Tantalizingly, there appeared to be two main strategies: (1) having many indirect interactions with other corals (through shared symbionts) on a gradient ranging through all of the generalist options, versus (2) having few indirect interactions with other corals and associating in a tight specialist-specialist interaction (Fig. 3a). Notably, there were no obligate relationships between a single coral species and a single *Symbiodinium* type at the global level.

**Table 1:** Basic measures of coral-*Symbiodinium* communities at reef, ocean basin, and global scales.

Country	Community	Coral	Sym	Asym	Edge	Conn	Nest	N*_1_	N*_2_	Refs
Australia	Curaçao Island	34	8	0.62	55	0.20	16.24	−0.05	−0.27	1
Australia	Feather Reef	74	17	0.63	125	0.10	20.96	1.43***	0.55**	1
Australia	Heron Island	72	35	0.35	118	0.05	17.28	3.16***	0.93***	5
Australia	One Tree Island	10	14	−0.17	21	0.15	9.93	−0.22	−0.31	1
Australia	Rib Reef	73	19	0.59	100	0.07	16.89	2.04***	0.81**	1
Australia	Western Australia	20	28	−0.17	55	0.10	12.68	0.34	0.10	2
Bahamas	Exuma Islands	16	15	0.03	28	0.12	10.07	−0.03	−0.14	4
Barbados	Eastern Caribbean	30	28	0.03	81	0.10	20.20	1.01***	0.51**	1
Belize	Carrie Bow	39	33	0.08	74	0.06	10.21	0.90***	0.48*	2
Japan	Zamami Island	51	15	0.55	73	0.10	15.06	0.98***	0.41*	1
Mexico	La Paz	11	10	0.05	34	0.31	36.75	0.15	−0.06	2
Mexico	Puerto Morelos	31	20	0.22	59	0.10	17.32	0.93***	0.47*	2
Tanzania	Banda Kuu	56	24	0.4	126	0.09	16.90	0.81***	0.12	1
Tanzania	Bawe	29	15	0.32	64	0.15	18.00	0.23	−0.06	1
Tanzania	Changuu	34	20	0.26	70	0.10	11.00	0.09	−0.23	1
Thailand	Cape Panwa	63	16	0.59	171	0.17	40.17	1.30***	0.46***	1
Thailand	Hae	54	11	0.66	84	0.14	1.70	−0.85	−0.88	1
Thailand	Phiphi Don	76	10	0.77	144	0.19	10.31	−0.40	−0.55	1
Thailand	Phiphi Lae	53	12	0.63	119	0.19	21.36	0.18	−0.18	1
Thailand	Racha	51	14	0.57	90	0.13	3.82	−0.66	−0.74	1
Thailand	Similan	54	15	0.57	102	0.13	7.53	−0.35	−0.53	1
United States	Florida Keys	13	10	0.13	33	0.25	41.36	0.62**	0.31	5
United States	Oahu	21	18	0.08	39	0.10	8.54	−0.10	−0.26	1
United States	US Virgin Islands	9	10	−0.05	29	0.32	51.19	0.54**	0.31	1
C. Indo-Pacif.		164	67	0.42	355	0.03	25.53	7.02***	1.95***	8
W. Indo-Pacif.		168	50	0.54	512	0.06	31.92	3.91***	1.11***	1
Trop. Atlantic		46	57	−0.11	176	0.07	18.73	1.61***	.76***	9
Global	All data	313	174	0.29	1060	0.02	22.81	9.73***	2.14***	21
Global	Well-sampled	54	106	−0.33	381	0.07	14.86	1.05***	.46***	21

Coral = number of coral species; sym = number of *Symbiodinium* types; asym = web asymmetry; edge = number of edges or interactions; conn = connectance; nest = nestedness (NODF); N^*^_1_ =  relative nestedness (model 1); N^*^_2_ =  relative nestedness (model 2); refs = number of references. Significance values:

*** <.001;

** <.01;

* <.05.

**Figure 4:**
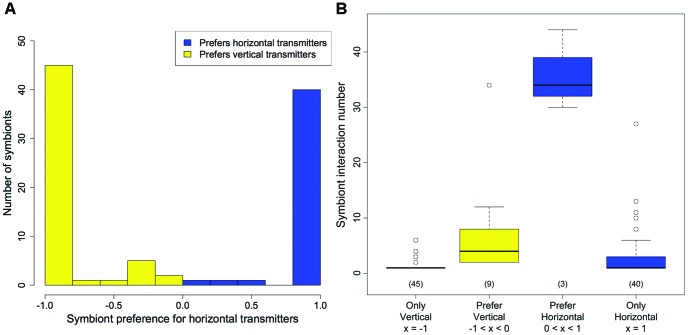
The relationship between *Symbiodinium* interaction numbers and preference for horizontally or vertically transmitting corals. (a) Symbiont preferences for (positive values) or against (negative) horizontally transmitting corals versus vertically transmitting corals. Zero indicates no preference, while 1 and −1 indicate coral hosts are only horizontal or vertical transmitters, respectively. (b) From left to right, symbionts which only interacted with vertical transmitters, interacted preferentially with vertical transmitters, interacted preferentially with horizontal transmitters, or only interacted with horizontal transmitters (x = k_diff_). Numbers in parentheses indicate sample size in each group.

The mean number of interactions for a coral’s associated *Symbiodinium* was correlated with that coral’s transmission mode (see partition in Fig. 3a). Corals that interact with specialist symbionts on average (symbionts with fewer than ∼14 coral hosts) were almost always vertical transmitters, while corals that interact with generalist symbionts on average (symbionts with more than ∼14 coral hosts) were almost always horizontal transmitters (Wilcoxon; W  = 377, m_vert_  = 9.11, m_horiz_  = 21.81, n_vert_  = 11, n_horiz_  = 35, p  = 2.14 * 10^−6^, two-tailed). We explored the interaction preferences for *Symbiodinium* in the global network by comparing their realized and expected number of interactions with horizontally transmitting corals based on proportion of horizontal transmitters in the community. *Symbiodinium* types interacted with either horizontal or vertical transmitters much more than expected, and most symbionts interacted with only one group (Fig. 4a). Indeed, 46% of all *Symbiodinium* types in the dataset were found exclusively in vertical transmitters and 41% in horizontal transmitters. Generalist symbionts were much more likely to interact with both horizontal and vertical transmitters (Fig. 4b), while symbionts that were found only in horizontal or vertical transmitters were specialists. Vertically transmitting corals and horizontally transmitting corals had no appreciable difference in their mean number of interactions, despite the differences in their symbionts’ mean number of interactions (Wilcoxon; W  = 139, m_vert_  = 8.91, m_horiz_  = 6.77, p  = .17, two-tailed).

We explored a few common, unweighted network metrics to compare coral-*Symbiodinium* networks to other mutualistic networks (Table 1; see methods). The global network of well-sampled corals species had few realized interactions relative to the number of potential interactions (connectance  = .07), had many more symbiont types than host species (web asymmetry = −.33), and was significantly nested (N^*^_1_ = 1.07, N^*^_2_ = .47, see Table 1). The ocean basin networks were also poorly connected and significantly nested, but the central and western Indo-Pacific had more coral species than symbiont types. Most reef-level networks (87.5%) had more coral (animal) species than *Symbiodinium* (plant) types, which, like animal-dominated pollination networks, seems to differ from many seed-dispersal and ant-myrmecophyte networks [33]. Reef-level nestedness values (a measure of order) were not significant when compared to nestedness values generated with null models 1 and 2. Only 46% of reefs were significantly nested under null model 1 and 33% were significantly nested under null model 2.

## Discussion

We explored a global compilation of coral-*Symbiodinium* interactions [34], and found that both corals and *Symbiodinium* were composed of many specialists and few generalists. Further, we found that symbiont transmission mode was correlated with mean *Symbiodinium* partner specificity, and not coral specificity. Communities were generally partitioned between vertically transmitting corals and their symbiont partners.

Coral species with the potential to interact with functionally diverse *Symbiodinium* assemblages are hypothesized to be more responsive to global changes, i.e., the Adaptive Bleaching Hypothesis. Mutualistic network studies in other systems, such as pollination and seed-dispersal communities, find consistent network characteristics and correlations with resilience. In particular, generalists should be more resilient to disturbances than specialists. Thus, interpreting our results under the assumption that the ability to interact with functionally diverse *Symbiodinium* assemblages is beneficial, or with previous network findings in other ecosystems, leads to the same conclusion. Such patterns would suggest that *Pocillopora damicornis* and *Stylophora pistillata*, the two most generalist coral species in the global network, would be more resilient species and communities with many generalist species would be the most resilient.

However, it is also possible that the high degree of intimacy in these symbioses maximizes fitness through the co-evolution of high-fidelity partners [35]. Tight functional integration may increase the range of environmental conditions in which a partnership is stable and stress resistant. Recent evidence [36] links generalist corals, such as *Acropora* and *Pocillopora*, to greater environmental sensitivity than specialists [37], [38], and suggests that corals with symbiotic specialization (e.g., massive *Porites*; [39]) may be more resilient to contemporary stressors [40]. Vertical symbiont transmission also results in lower variation in partnerships between generations, and greater potential for co-evolution and increasing levels of symbiotic integration [26]. Corals that are both specialists and vertical transmitters, such as *Porites lobata* and *Montipora digitata*, would then have more resilient populations.

Community resilience is also dependent on symbiont functional characteristics. For example, *Symbiodinium* types vary in their effects on coral growth rate [18], [19], carbon fixation and photosynthate transfer [8], [19]. Further, a coral’s ability to cope with environmental stressors, such as temperature anomalies, is related to symbiont identity [12], [14], [15]. Thus, *Symbiodinium* vary in the benefit they provide to their hosts depending on the community composition and environmental conditions. Unfortunately, not enough is known about symbiont functional characteristics to include this in our analysis [9]. Nonetheless, differences between *Symbiodinium* types and unique coral-*Symbiodinium* pairings are certain to make coral resilience a function of both the number and diversity of symbiont partners. While we have quantitatively described the association potential of numerous coral species, definitively answering the resilience question is beyond the scope of our study and requires further investigation into functional response at *Symbiodinium* type level.

Our results provide evidence that contrasts with previous thoughts about coral-*Symbiodinium* relationships. First, many authors have suggested that corals are strict specialists due to the evidence that corals at local scales seem to interact with only one symbiont type [40, 41, but see [42]). Our results show that coral species have the potential to interact with many types (Fig. 3) and local specificity may be due to sampling methodology, or the limited spread of symbiont types (suggested by [13]). Notably, we found no obligate relationships between a coral species and a symbiont type at global scales. *Porites rus* was the only well-sampled coral to host a single symbiont type at global scales, but C15, its symbiont partner, has many alternative hosts. Second, previous authors have also suggested that vertically transmitting corals may be more specialist [13] and we provide evidence that coral specificity is unrelated to transmission mode (although it is related to symbiont specificity) (Fig. 3). Third, Wicks *et al*. [43] suggest that vertically and horizontally transmitting corals share symbionts, but it appears that symbionts are strongly partitioned (Fig. 4). Finally, previous studies suggest that vertically and horizontally transmitting corals have equivalent symbiont diversity at local scales [42]. In contrast, we find that there are many more recognized *Symbiodinium* types transmitted in vertically transmitting corals (Fig. 4a). Our results are consistent with the findings of Stat et al [44] on a local scale in the southern Great Barrier Reef, perhaps because vertical transmission promotes diversification via isolation and maintained associations. These results suggest that future research should discern which patterns represent true barriers to association and which reflect biogeographic distributions (and thus the difference between a global or local perspective).

Our results are a product of a still incomplete understanding of coral-Symbiodinium interactions and symbiont diversity. *Symbiodinium* types have been described using several markers (nrDNA, rSSU, PLSU, ITS-1 and 2) and methodologies (DGGE, RFLP, SSCP, bacterial cloning, and direct sequencing) [13]. The range of methods makes it difficult to create a comprehensive phylogeny within clades, and the identity, number, and ecological meaning of types is sometimes disputed [45], [46], 47. Researchers generally recognize DGGE ITS-2 types as identifying dominant *Symbiodinium* types, and our methods were chosen according to this interpretation. While our study provides new insights on data already collected, the next step is to use detailed high-resolution datasets to explore the sensitivity of our results to more detailed interaction data. Indeed, although we have the ability to create reef and ocean basin networks with GeoSymbio, we were forced to restrict most of our analyses to the global network of well-sampled coral species. Sampling intensity is variable between reefs and ocean basins and many network analyses are sensitive to species diversity or missing links.

Further studies with rigorous sampling designs will provide the detail necessary to explore the conservation implications of these patterns. Our dataset is an aggregation of coral-*Symbiodinium* interactions compiled across time and space [34] and from different researchers employing a variety of methodological approaches. However, we focus our analyses on the most well-sampled coral species. Our qualitative results differ only when severely undersampled species are included, and only affect the partition found in Fig. 3a. All other qualitative results are robust and increasing the number of samples or references needed for data inclusion only strengthens the observed patterns. However, certain network metrics are sensitive to species diversity, rare interactions, and other phenomena [48], [49], [50] and future work is needed to include more sampling information and construct specific experiments designed to test network hypotheses. Based on these early results, the combination of network analyses with ecological, evolutionary, and environmental data appears to be a powerful quantitative approach for analyzing relationships between corals and their symbiotic partners.

## Methods

We studied coral-*Symbiodinium* networks to quantify the specificity of corals and *Symbiodinium* at reef, ocean basin, and global scales, and to explore how specificity correlates with transmission mode. We used data from GeoSymbio, a database of interactions between *Symbiodinium* and their animal hosts from 79 publications between 1982 and 2010 [34]. Interaction records were filtered to meet several criteria such that the final subset of data was aggregated from 21 publications with data collected between 1998 and 2009. First, only interaction records between hard corals (order *Scleractinia* identified at the genus and species level) and *Symbiodinium* (identified by subtype, which corresponds to differences in the Internal Transcribed Spacer Region 2 [ITS2] sequence) were included. Second, we restricted methodology to denaturing gradient gel electrophoresis (DGGE), which identifies the dominant *Symbiodinium* sequence within a coral sample [51]. Third, we removed any aquarium records. Finally, records were removed if *Symbiodinium* sequence data were unavailable in GenBank, and together, these criteria minimized methodological differences.

We created coral-*Symbiodinium* networks at reef, ocean basin, and global scales to study interaction patterns. Coral species and *Symbiodinium* types were connected in a community’s interaction network if a coral species had been recorded as hosting a *Symbiodinium* type. Mutualistic interaction networks typically represent interactions between species, rather than genera or clades, and we follow this convention as closely as possible given the current taxonomy of *Symbiodinium*, where ITS2 types are thought to be most closely related to the species designation. As sampling intensities were not recorded within all references, we chose to analyze unweighted networks, which tracks the presence or absence (1 or 0) of interactions, rather than weighted networks, which additionally identifies the strength of interactions.

The interaction data were filtered to create reef-level or local networks. Geographical information for coral-*Symbiodinium* type associations in GeoSymbio followed a hierarchical framework modified from the Ocean Biogeographic Information System data schema (v1.1). To standardize community sampling effort based on the available data for sub-ocean basin scale networks, sample location levels were examined by A) state-region, B) subregion, and C) locale in the following fashion. Interaction data were included in our analyses if more than 30 interaction records were available at the state-region level, and each of the lower levels were subsequently examined if 30 interactions were available. Thirty interaction records were chosen as a lower threshold for community sampling because of a natural break between poorly sampled and well-sampled communities within GeoSymbio and to correspond to other mutualistic networks. Western Australia and the Exuma Islands, Bahamas were the only maximally-reduced state-region communities. Ocean basin networks were constructed by combining data for only the adequately sampled reef-level networks. The spatial boundaries for the western Indo-Pacific, central Indo-Pacific, and tropical Atlantic are consistent with the biogeographical realms in [52]. La Paz, Oahu, and Zamami Island are not included in the ocean-level analysis due to the lack of data for the associated biogeographical realms.

We created global interaction networks by aggregating only the adequately sampled reef-level networks in GeoSymbio. The first global network included all of the data from the adequately sampled reef-level networks, while a second global network only included data from well-sampled corals. For inclusion in the well-sampled global interaction network, coral species needed to be sampled at least ten times from at least three different publications to minimize artifacts associated with sampling bias. *Symbiodinium* types needed to be recorded in at least one of the well-sampled coral species, as poorly-sampled corals could provide an inadequate picture of their symbiont populations, while rare *Symbiodinium* in well-sampled corals reflect an actual interaction trend. Despite the likelihood that increased sampling effort would increase the number of interactions reported, and that *Symbiodinium* diversity analyses are a much needed area of research globally, many specialist taxa are particularly well sampled and still have very few associated symbionts (see *Montipora digitata* and *Porites cylindrica*, Fig. 3b), displaying a trend rather than an artifact. Following the data filtering, we quantified the specificity of coral species for *Symbiodinium* types using descriptive statistics, and examined correlations with previously identified transmission mode (sensu [24]).

The specificity of coral hosts and their symbiont partners was further explored by clustering corals according to their degree (number of interactions) and the mean or median degree of their symbiont partners. We clustered corals using the k-means clustering algorithm, where the number of clusters, *k*, is predefined and the algorithm locates cluster means by optimizing the within cluster sum of squares relative to the across cluster sum of squares. The optimal number of clusters was selected conservatively by determining when less than 10% variance, or between cluster sum of squares to total sum of squares, was explained by an additional cluster. Cluster identity was robust to the use of mean or median symbiont degree. Correlations between coral transmission mode [24] and coral interaction number, and mean symbiont interaction number were tested using the Wilcoxon rank-sum test.

The partitioning between horizontally and vertically transmitted symbionts was tested using a modified network assortment metric [53]. Assortment measures the degree to which members of distinct groups interact with one another more or less than expected. For each symbiont in the well-sampled global network, we determined its total number of interactions, *k*, its total number of interactions with horizontally transmitting corals, *k_h_*, and the proportion of horizontally transmitting corals in the community, *p_h_*. The expected number of interactions with horizontally transmitting corals given random interactions would then be
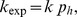
and the difference between the expected and realized number of interactions is



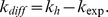



Symbionts interact with horizontally transmitting corals more than expected if *k_diff_* is positive, and interact with vertically transmitting corals more than expected if *k_diff_* is negative. We then normalize *k_diff_* based on the maximum or minimum possible value so that *k_diff_* ranges from 1 (when symbionts only interact with horizontally transmitting corals and *k_h_* = *k*) to −1 (when symbionts only interact with vertically transmitting corals and *k_h_*  = 0). Thus,
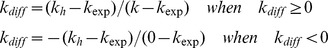



Coral-*Symbiodinium* network metrics were explored to determine whether general trends exist and how those trends compare to other mutualistic networks. Mutualistic interaction networks are characterized by properties that generally hold across wide geographic scales and interaction types (reviewed in [27], [28]). These include: significantly more species in one guild than the other (web asymmetry), a low number of realized interactions (connectance), high levels of nested organization (nestedness, see below), and many specialists versus few generalists. The consistency of these patterns has led to several hypotheses for their existence, including population sizes, optimal behavior, trait matching and barriers, and phylogenetic relationships [28]. Most importantly, these traits may relate to the resilience - the ability to withstand perturbations - of a community under certain stressors [29], [30], [31].

We calculated web asymmetry, connectance, and nestedness, and created degree distributions for each network [53]. Web asymmetry identifies networks with more coral types, *c*, than *Symbiodinium* types, *s*, and is equal to the number of coral species minus the number of *Symbiodinium* types, divided by the total number of types and species (*(c − s)/(c + s)*). Positive web asymmetry values indicate more coral species, while negative values indicate more *Symbiodinium* types. Connectance is the proportion of realized interactions or links, *l*, out of the total potential interactions, or the number of interactions divided by the number of coral species times the number of *Symbiodinium* types (l/(c * s)). Degree distributions are created by identifying the relative frequency, *f_d_*, of species with degree *d*, or a total of *d* interactions.

Nestedness is a measure of order in a community, where high levels of order indicate that the partners of specialists are subsets of the partners of generalists. We calculated nestedness using the overlay and decreasing fill (NODF) method via the *nestednodf()* function in the R package vegan ([54], using method = “NODF2”). Nestedness values range between 0 and 100, with greater values indicating greater nestedness. Nestedness values for the actual network were compared to null model expectations [55]. The first null model holds the number of links in a network constant (connectance), while the second assumes the probability of two species interacting is equal to the average of their respective probabilities of interaction. We ran 1000 replicates for each null model and calculated relative nestedness to compare nestedness between networks [56]. Relative nestedness is defined as
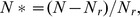
Where *N* is the NODF2 nestedness value for the actual network and *N_r_* is the mean NODF2 nestedness value across all null model replicates.

## Supporting Information

Table S1 Well-sampled coral species from the central Indo-Pacific and western Indo-Pacific. “Trans” = transmission mode; “Syms” = number of symbiont partners; “CIP” = found in central Indo-Pacific; “WIP” = found in western Indo-Pacific; “TA” = found in tropical Atlantic; “Samp” = number of samples; “Refs” = number of references. Transmission codes are “H” = horizontal, “V” = vertical, and “M” = mixed.
(TIFF)

Table S2 Well-sampled coral species from the tropical Atlantic. “Trans” = transmission mode; “Syms” = number of symbiont partners; “CIP” = found in central Indo-Pacific; “WIP” = found in western Indo-Pacific; “TA” = found in tropical Atlantic; “Samp” = number of samples; “refs” = number of references. Transmission codes are “H” = horizontal, “V” = vertical, and “M” = mixed.
(TIFF)

Data S1 **GeoSymbio data.** Data from GeoSymbio (https://sites.google.com/site/geosymbio/) used for analyses.
(CSV)

Data S2 **Transmission mode information.** Transmission mode information from [24].
(CSV)

Schema S1 **Schema for GeoSymbio data (Data S1).**
(CSV)

References S1 **References for GeoSymbio data (Data S1).**
(CSV)
