# Supplementary figures and images for: Transmission Mode Predicts Specificity and Interaction Patterns in Coral-Symbiodinium Networks
Source: PLoS One. 2012 Sep 18;7(9):e44970. doi: 10.1371/journal.pone.0044970 (PMC3445617; doi:10.1371/journal.pone.0044970)

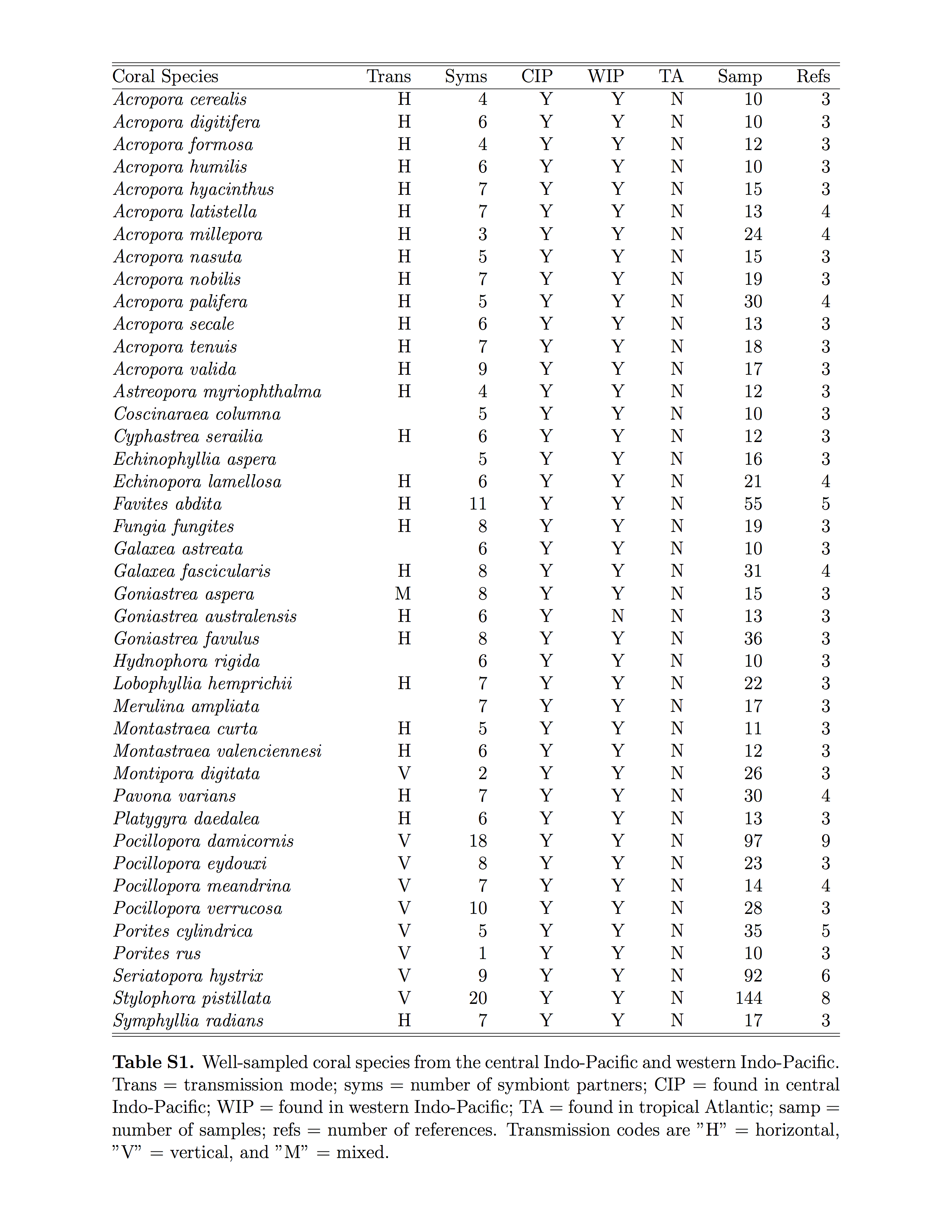

Supplement: Table S1 — Well-sampled coral species from the central Indo-Pacific and western Indo-Pacific. “Trans” = transmission mode; “Syms” = number of symbiont partners; “CIP” = found in central Indo-Pacific; “WIP” = found in western Indo-Pacific; “TA” = found in tropical Atlantic; “Samp” = number of samples; “Refs” = number of references. Transmission codes are “H” = horizontal, “V” = vertical, and “M” = mixed. (TIFF) [file pone.0044970.s001.tiff]

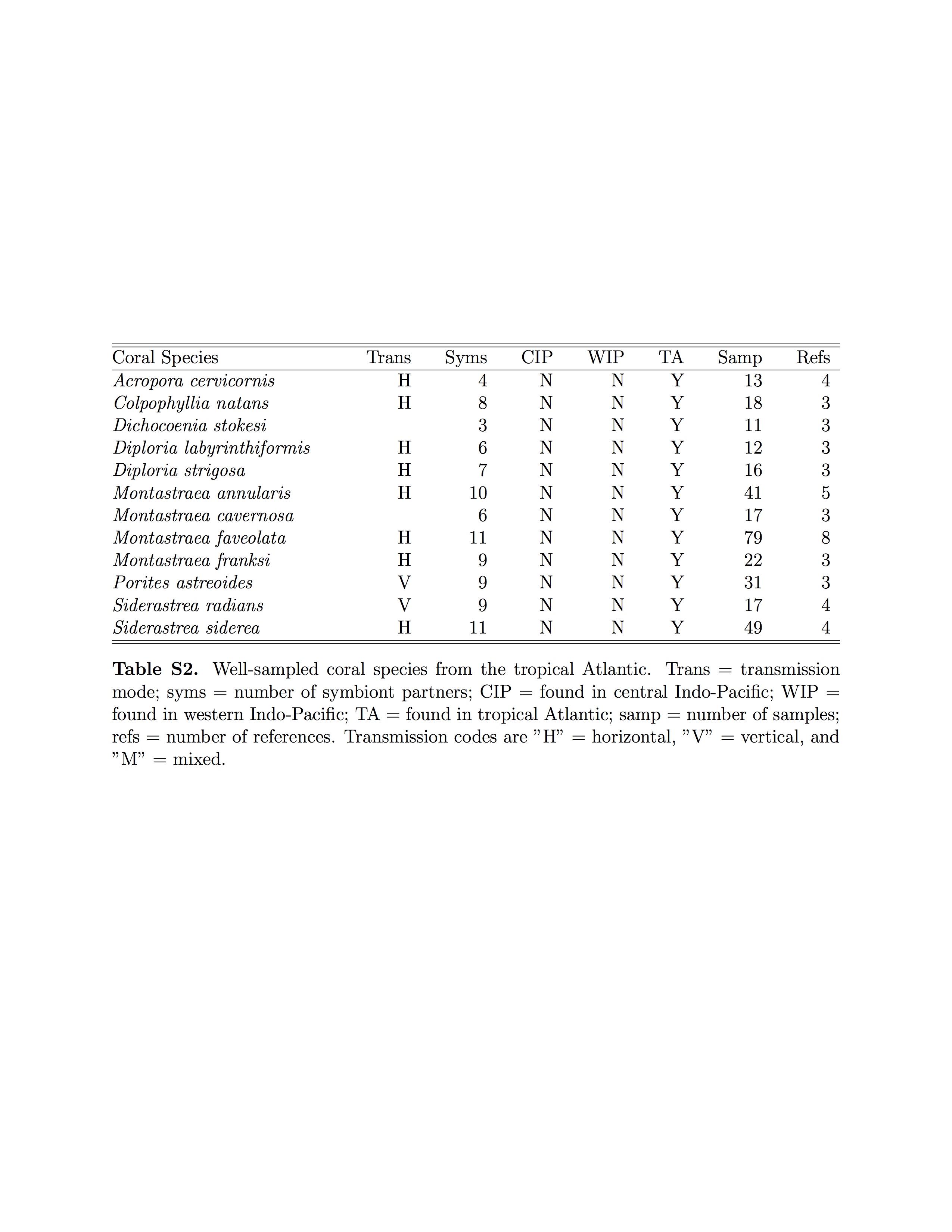

Supplement: Table S2 — Well-sampled coral species from the tropical Atlantic. “Trans” = transmission mode; “Syms” = number of symbiont partners; “CIP” = found in central Indo-Pacific; “WIP” = found in western Indo-Pacific; “TA” = found in tropical Atlantic; “Samp” = number of samples; “refs” = number of references. Transmission codes are “H” = horizontal, “V” = vertical, and “M” = mixed. (TIFF) [file pone.0044970.s002.tiff]
